# Update on Anaplastic Thyroid Carcinoma: Morphological, Molecular, and Genetic Features of the Most Aggressive Thyroid Cancer

**DOI:** 10.1155/2014/790834

**Published:** 2014-08-21

**Authors:** Moira Ragazzi, Alessia Ciarrocchi, Valentina Sancisi, Greta Gandolfi, Alessandra Bisagni, Simonetta Piana

**Affiliations:** ^1^Pathology Unit, IRCCS-Arcispedale Santa Maria Nuova, Viale Risorgimento 80, 42123 Reggio Emilia, Italy; ^2^Laboratory of Translational Research, Research and Statistic Infrastructure, Arcispedale S. Maria Nuova-IRCCS, 42123 Reggio Emilia, Italy

## Abstract

Anaplastic thyroid carcinoma (ATC) is the most aggressive form of thyroid cancer. It shows a wide spectrum of morphological presentations and the diagnosis could be challenging due to its high degree of dedifferentiation. Molecular and genetic features of ATC are widely heterogeneous as well and many efforts have been made to find a common profile in order to clarify its cancerogenetic process. A comprehensive review of the current literature is here performed, focusing on histopathological and genetic features.

## 1. Introduction

Anaplastic thyroid carcinoma (ATC) represents the most aggressive extreme of the clinical spectrum of thyroid epithelial neoplasms, being one of the most lethal human tumors.

It constitutes less than 5% of clinically recognized thyroid malignancies but it accounts for more than half of the deaths for thyroid cancer, with a mortality rate that is over 90% and a mean survival of six months after the diagnosis.

It is defined by the WHO as a highly malignant tumor wholly or partially composed of undifferentiated cells that retain features indicative of an epithelial origin, on immunohistochemical or ultrastructural ground [1]. It usually affects elderly people, with a mean age in the mid-60s, and shows a female predominance [1].

In this review we tried to summarize the current knowledge on ATC from both morphological and biological points of view.

## 2. Morphological Features

**Figure 1:**
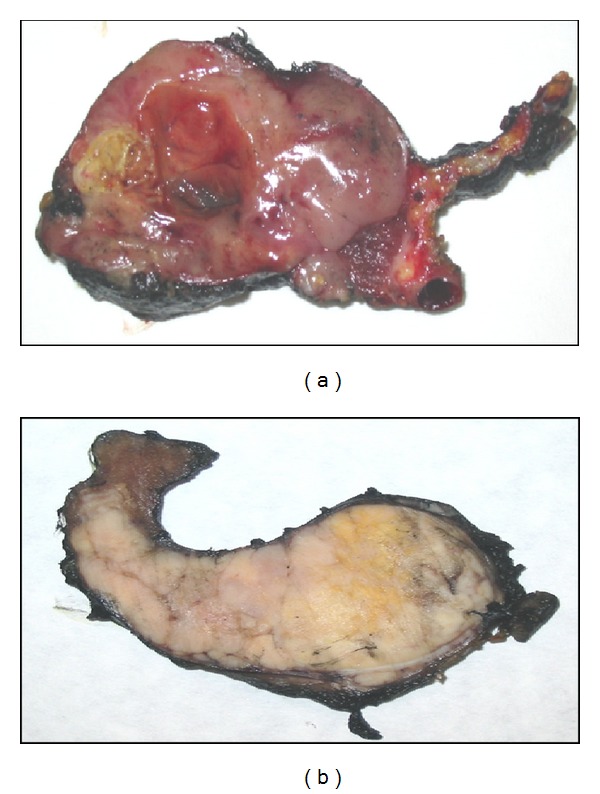
Grossly, ATC shows a diffusely infiltrative pattern of growth. The cut surface can be brownish (a) or whitish (b); in both specimens discrete yellowish areas of necrosis are evident.

Grossly, ATC is well recognized as a large, necrotic, and hemorrhagic mass that is typically widely invasive, often replacing most of the thyroid gland parenchyma with infiltration of the surrounding soft tissue and adjacent structures of the neck (Figures 1(a) and 1(b)).

The morphological spectrum depends on the admixture of three main histological patterns: spindle cell, giant cell, and squamoid [2–4]. These patterns often coexist and are not predictive of patients' outcome but are historically used to group ATC in major histological categories and to define their main differential diagnoses. The histological categories are sarcomatoid and epithelioid-squamoid.

The small cell category, that was included in older classification of ATC, is no longer considered, as it comprised cases of bona fide lymphomas, medullary carcinomas, and insular carcinomas [2, 3, 5].

Common features to all patterns of ATC are hypercellularity, large foci of necrosis, marked invasiveness, and angiotropism with a tendency to infiltrate medium-sized veins and arteries, replacing their muscular wall [2, 3].

For diagnostic purposes, fine needle aspiration biopsy (FNAB) is an important tool and can provide a correct diagnosis of ATC in up to 84% of cases [6].

**Figure 2:**
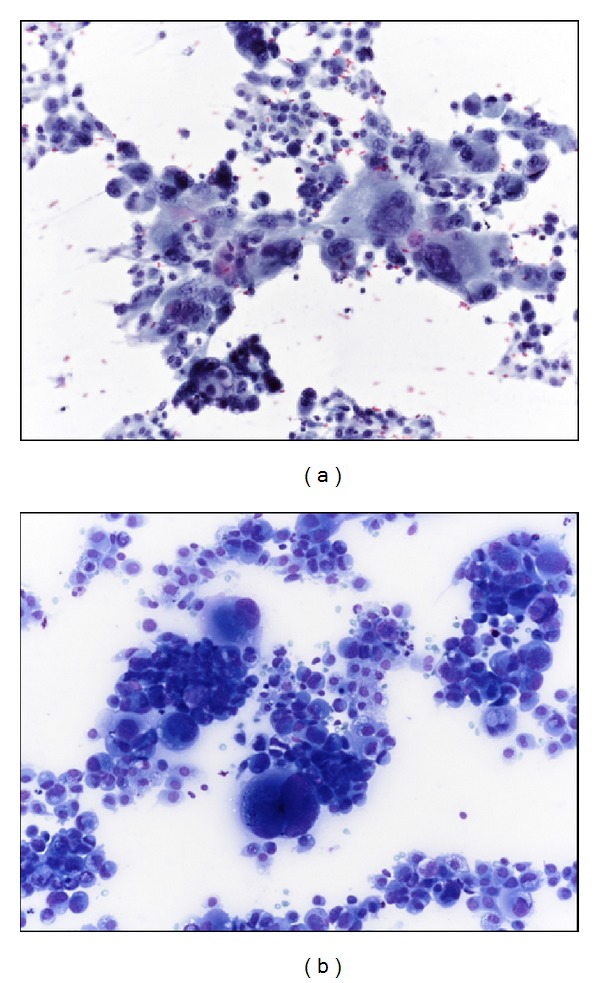
FNAB smears are usually made up of polymorphic neoplastic cells in a dirty necrotic background ((a) Papanicolaou stain, (b) May-Grunwald Giemsa stain).

FNAB smears are usually composed of a pleomorphic cellular population in a necrotic background (Figures 2(a) and 2(b)). The tumor cells are bizarre, oval to spindle-shaped, dyscohesive elements showing anisocytosis, and irregular sometimes multiple nuclei, perfectly reflecting the sarcomatoid or epithelioid histological morphology.

### 2.1. Sarcomatoid Category

#### 2.1.1. Histology

Anaplastic thyroid carcinomas with sarcomatoid appearance are characterized by spindle cells and giant cells, the most frequent patterns seen in ATC. In fact, spindle and giant cells have been found, alone or in combination, in at least 50% of cases reported by Carcangiu and colleagues [2].

**Figure 3:**
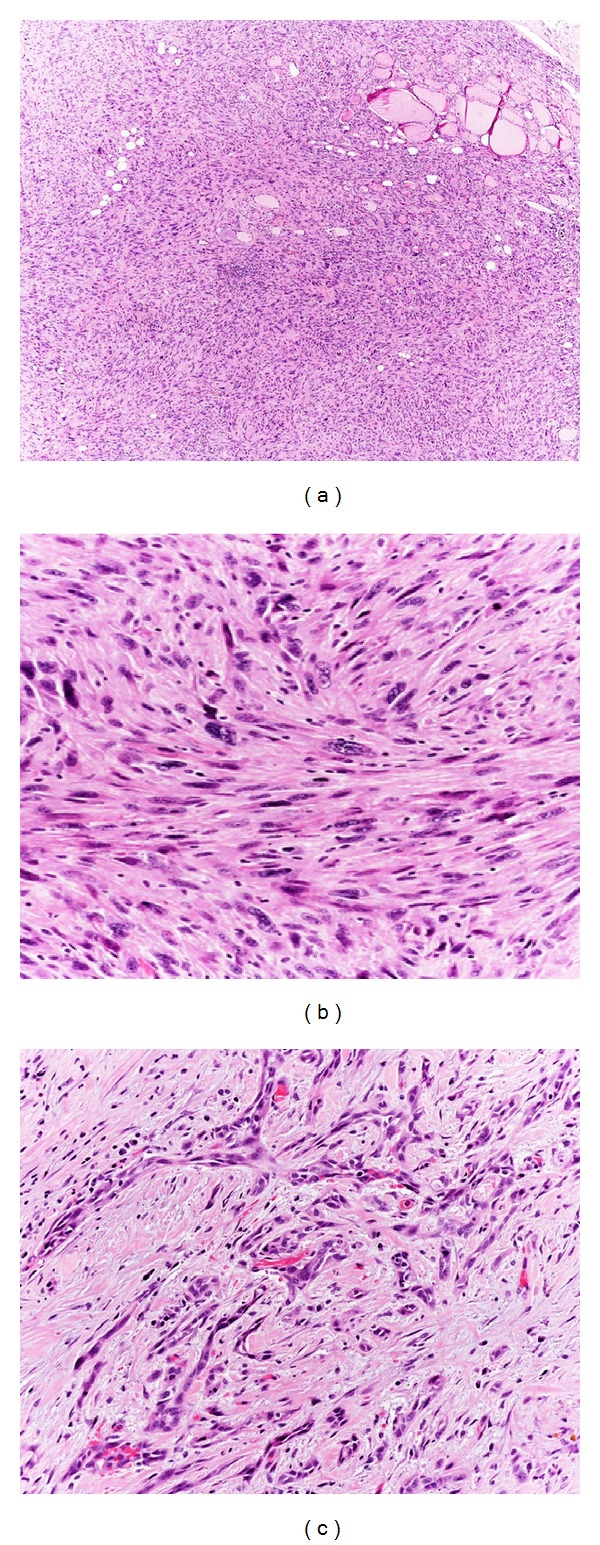
(a) In sarcomatoid ATCs, neoplastic cells are morphologically indistinguishable from a primary sarcoma. At higher power view (b), spindle cells are pleomorphic and show a storiform pattern of growth. (c) Anastomosing cords of neoplastic cells resembling neoplastic vessels are the dominant features in this case.

*Spindle cells* show a fascicular or storiform pattern of growth, indistinguishable from a true sarcoma (Figures 3(a) and 3(b)). These neoplasms are generally well vascularized often resulting in a hemangiopericytoma-like pattern or forming anastomosing channels lined by tumor cells, resembling an angiosarcoma (Figure 3(c)). An odd variation on the theme of the spindle cell form is the paucicellular variant [7, 8]. This infrequent entity was first described by Wan et al. in 1995 as a peculiar subtype of ATC with gross and histological features closely mimicking Riedel's thyroiditis [7]. It is characterized by low cellularity with striking degree of fibrosis and hyalinization, presence of spindle cells resembling fibroblasts or myofibroblasts, absence of obvious nuclear atypia, and sprinkling of lymphocytes. Features allowing a diagnosis of ATC are (1) presence of coagulative necrosis with ghost shadows of preexisting blood vessels, (2) recognition of scattered atypia and mitosis in more cellular areas at the periphery of the fibrosis, (3) detection of blood vessels obliterated by neoplastic spindle cells, and (4) positivity for epithelial markers [7].

**Figure 4:**
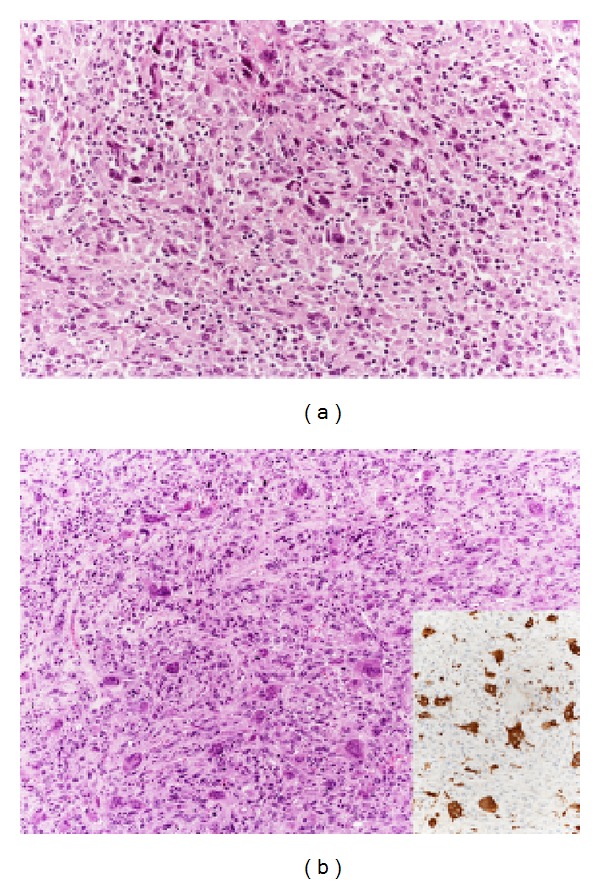
Neoplastic giant cells are characterized by deep pleomorphism, with bizarre multiple hyperchromatic nuclei (a). They differ from reactive osteoclast-like giant cells (b) that show bland cytological features and are typically immunoreactive for CD68-KP1 (inset).

*Giant cells* are characterized by deep pleomorphism, having bizarre sometimes multiple hyperchromatic nuclei, abundant eosinophilic cytoplasm, and a plump, oval, or round shape (Figure 4(a)). They are typically interspersed among smaller mononuclear tumor cells with similar cytoplasmic features showing a solid architecture. The formation of alveolar, pseudoglandular, or pseudovascular structures can also be seen, probably due to an artefactual separation of the cells. The cytoplasm of the tumor cells can sometimes assume a clear or granular appearance simulating a clear cell or an oncocytic carcinoma, respectively; the presence of striking pleomorphism, high mitotic activity, and necrosis is strongly suggestive for ATC [2].

*Osteoclast-like multinucleated giant cells* are occasionally present and could be prominent, resembling similar tumors described in breast and pancreas. Osteoclast-like multinucleated giant cells are known to be reactive elements of monocytic/histiocytic lineage, immunohistochemically positive for CD68-KP1 (Figure 4(b)) and apparently derived from histiocytoid mononuclear cells via cellular fusion [9]. They give the tumors an appearance reminiscent of giant cell tumor of bone and soft tissue.

*Huge inflammatory infiltrate* is often present, sometimes predominantly neutrophilic in type, giving the tumor an appearance resembling inflammatory variant of malignant fibrous histiocytoma.

*Heterologous elements*, such as bone, cartilage, and skeletal muscle, can also be found. Matrix formation with chondro- and osteosarcomatous differentiation has been reported in up to 5% of anaplastic carcinoma [10]. Rhabdomyosarcomatous appearance has also been described [2, 11]. Carda et al. reported two cases, in which the skeletal muscular differentiation was demonstrated by electron microscopy and immunohistochemistry with positivity for muscle-specific actin, desmin, myogenin, and MyoD1 [11].

#### 2.1.2. Differential Diagnosis

Sarcomatoid ATC closely simulates a large variety of soft tissue sarcomas. When a well-differentiated component is lacking and immunohistochemistry fails to demonstrate an epithelial differentiation, this distinction could be really difficult. Two characteristic histological features are helpful to differentiate sarcomatoid anaplastic carcinoma from a true sarcoma: the presence of angulated necrotic foci with neoplastic cells palisading around them as seen in glioblastoma of the central nervous system and the tendency of the spindle neoplastic cells to infiltrate the wall of large-sized veins and arteries [2].

It should be kept in mind however that primary sarcomas of the thyroid are indeed very rare so that it has been suggested that all sarcomatoid tumors of the thyroid gland should be regarded as ATC [12].

Primary sarcomas simulating a sarcomatoid ATC have been reported as case reports: fibrosarcoma [13], leiomyosarcoma [14], chondrosarcoma [15], osteosarcoma [16], and angiosarcoma (including epithelioid variant) [17, 18]. Metastases are possible as well and should be clinically ruled-out [19–21].

In addition, various spindle cell neoplastic and nonneoplastic thyroid lesions could simulate a sarcomatoid pattern and they should be taken into consideration by pathologist during diagnostic process. Differential diagnoses are described in Table 1.

### 2.2. Epithelioid-Squamoid

#### 2.2.1. Histology

**Figure 5:**
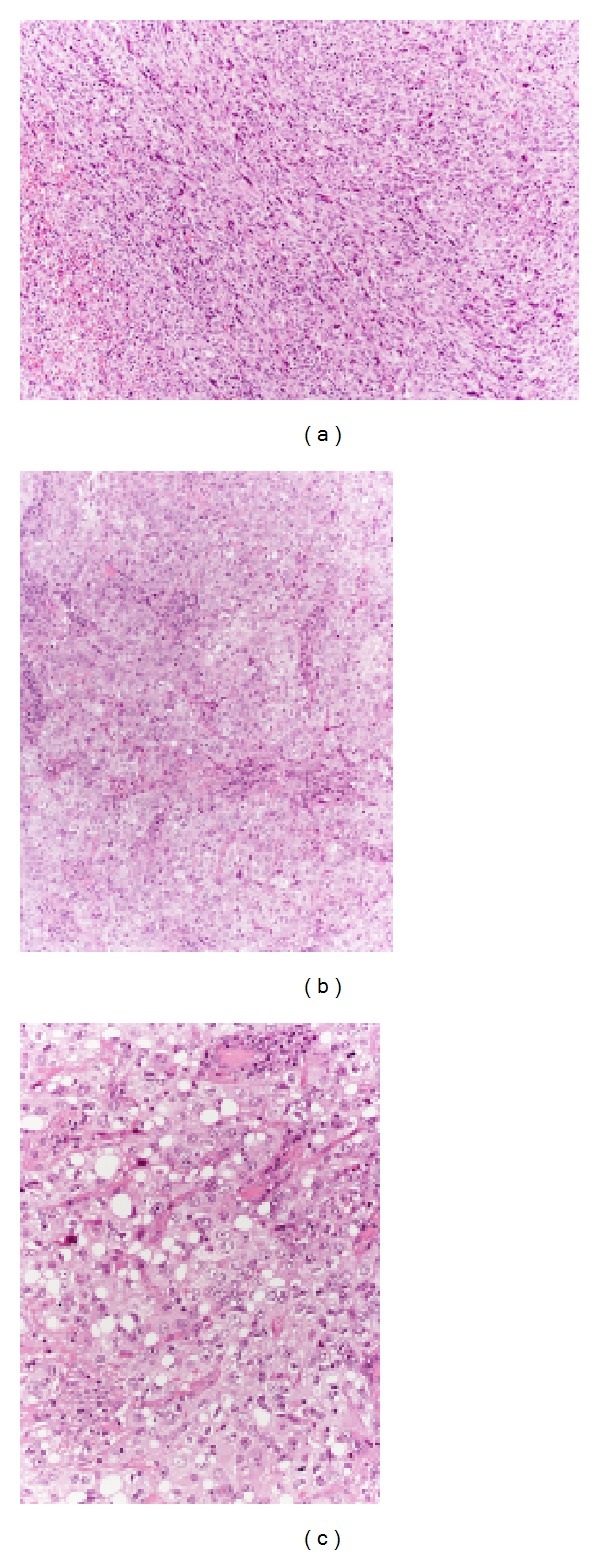
In the epithelioid-squamoid category, neoplastic cells show a solid (a) or nested (b) architecture. They are plump and have abundant cytoplasm with a variable degree of squamous differentiation (c).

Anaplastic thyroid carcinomas with epithelioid-squamoid appearance are histologically less heterogeneous than sarcomatoid tumors. They are characterized by polygonal cells with a clearly epithelial appearance, growing in solid nests, intermingled by desmoplastic stroma (Figures 5(a), 5(b), and 5(c)). Keratinization could be seen even if rarely. Squamoid pattern was present in about 20% of ATCs described in the largest series reported in literature [2, 4] and it is most frequently seen in combination with spindle and/or giant cells patterns.

Two peculiar variants of ATC belong to epithelioid-squamoid category and are described below.

*Anaplastic spindle cell squamous carcinoma* is a variant of ATC with both spindle cell elements and squamous islands with focal keratinization, similar to its counterpart described in the breast [22] and oropharynx [23, 24]. It was originally described by Bronner and LiVolsi as a unique subtype of ATC associated with the tall cell variant of papillary thyroid carcinoma (TCV PTC) [25]. In a recent series it was pointed out that this variant of ATC could clinically and histologically mimic a laryngeal squamous cell carcinoma. Therefore, caution is warranted in evaluating laryngeal squamous lesions in patients with known history of TCV PTC and without known risks factors for head and neck carcinogenesis [26].

*Lymphoepithelioma-like ATC* is a subtype of epithelioid-squamoid ATC characterized by histologic features similar to those of lymphoepithelioma of the nasopharynx and lymphoepithelioma-like carcinoma (LELC) of other sites [27]. It is composed of sheaths of epithelial cells in a rich inflammatory background including lymphocytes and some plasma cells. Tumor cells are immunoreactive for epithelial membrane antigen and keratin but are negative for thyroglobulin. Notably there is not association with EBV infection, as in LELC of organs that are not embryologically derived from primitive pharynx or foregut such as skin [28], urinary bladder [29], and uterine cervix [30].

#### 2.2.2. Differential Diagnosis

Pure squamous cells carcinoma of the thyroid is exceedingly rare and is listed as a separate entity in the WHO [1]. Its clinical presentation and behavior are the same of ATC. It is by definition not associated with other types of thyroid carcinoma.

**Figure 6:**
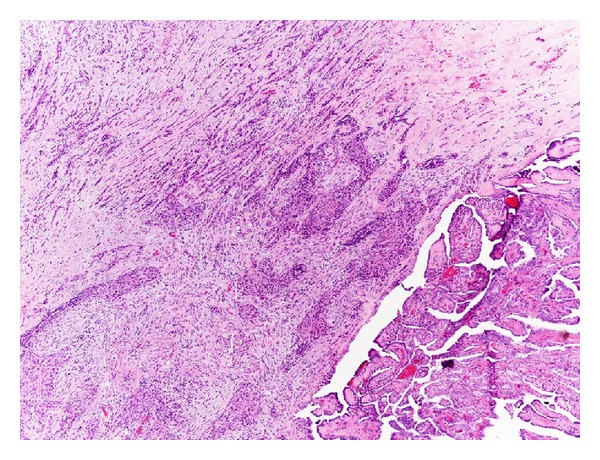
A significant rate of ATC is associated with WDTC. In this case, residual foci of papillary carcinoma are seen in the lower right corner but the main bulk of the tumor is composed of strands of squamoid atypical cells and spindle neoplastic elements.

When a thyroid tumor is almost composed of squamoid elements it could be also necessary to rule out a direct invasion from an upper airway primary or a metastatic process firstly from the lung. Careful clinical examination is the most important clue, particularly to exclude metastases. The search of a well-differentiated component, by extensive sampling of the surgical specimen, is also a helpful feature in identifying the tumor origin (Figure 6) and it is mandatory in these cases. Notably Toner et al. reported some cases of ATC with endotracheal presentation, showing metaplasia or atypical, probably regenerative, epithelial changes in the adjacent airway epithelium that could be easily misinterpreted as an in situ component [31].

In thyroid, squamous differentiation may be seen in other neoplastic settings, without the meaning of anaplastic transformation. Squamous differentiation can be present as a result of a metaplastic process in papillary carcinoma, most commonly in the diffuse sclerosing variant [32], in medullary carcinoma, in mucoepidermoid carcinoma, and in sclerosing mucoepidermoid carcinoma with eosinophilia [1]. Squamous differentiation is also present in most cases of “carcinoma showing thymus-like differentiation” (CASTLE), which is thought to arise either from ectopic thymus or remnants of branchial pouches [33, 34].

On the other hand, nonneoplastic squamous cells can be present as embryonic remnants in the thyroglossal duct or structures derived from the branchial pouch (e.g., thymic epithelium) and as squamous metaplasia in thyroiditis or as a reparative phenomenon following FNAB [35] and postradiation therapy.

Differential diagnoses are summarized in Table 2.

### 2.3. Immunohistochemical Features

ATCs show a variable immunophenotype. Immunoreactivity for cytokeratin is present in 40% to 100% of cases according to the different series [2, 36–38]. Vimentin is consistently present in the spindle cell component, whereas EMA and CEA are particularly expressed in the squamoid cells [2].

**Figure 7:**
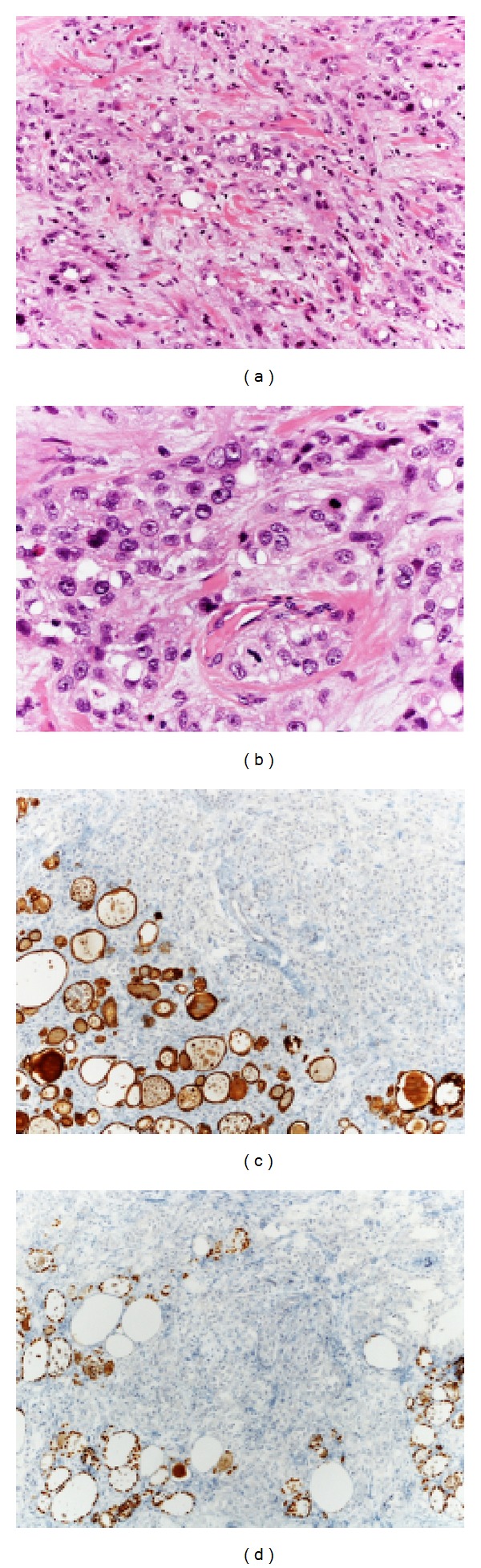
An ATC made up of pleomorphic epithelioid cells arranged in loosely cohesive nests; focally, intracytoplasmic vacuoles are present ((a), (b)). Immunohistochemical stains with keratin 7 (c) and TTF1 (d) highlight entrapped thyroid follicles.

Typically, ATC cells are not immunoreactive for thyroglobulin, calcitonin, TTF-1, or RET/PTC oncoprotein [36, 37, 39]. False positive reaction to thyroglobulin may result from nonneoplastic thyroid follicular cells entrapped in the tumor or diffusion from destroyed normal follicles (Figures 7(a), 7(b), 7(c), and 7(d)). PAX8 (also known as paired box gene 8) is a transcription factor expressed in nuclei of normal and neoplastic tissue of the thyroid, kidney, and female genital tract [40–42], being retained also in most sarcomatoid renal cell carcinomas. The few studies evaluating PAX8 staining of ATCs have had widely disparate results [40, 43, 44]. Nevertheless PAX8 seems to have a useful diagnostic role in specific settings, having been found in 79% of ATCs and in up to 92% of ATCs showing squamoid features, whereas it is negative in head and neck squamous carcinoma and lung carcinoma [40, 45].

Immunohistochemical features are summarized in Table 3.

## 3. Genetic Features

**Figure 8:**
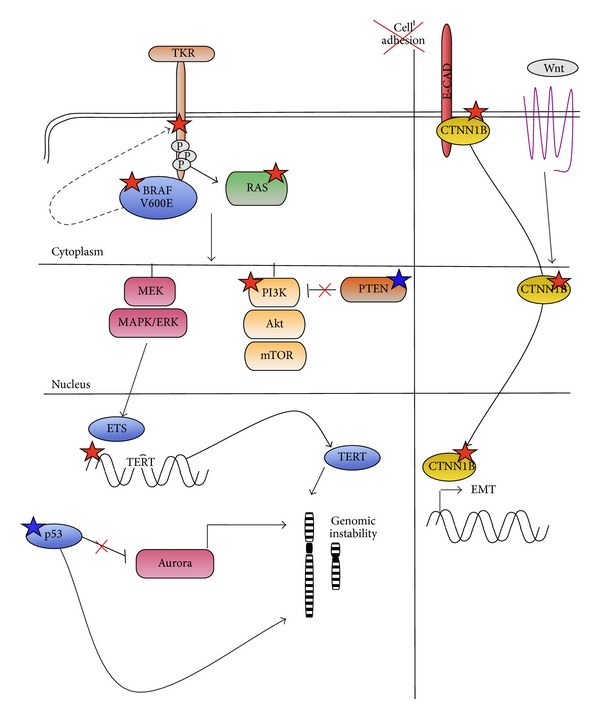
Schematic representation of molecular pathways altered in ATCs.

Even though ATC is a rare disease, a consistent amount of information is currently available on the genetic alterations that are most frequently associated with this tumor [46, 47] (Figure 8).

### 3.1. Somatic Gene Mutations

Mutations in the components of the principal oncogenic pathways (MAPK, PI3K, Wnt, etc.) have been described to occur with high frequency in ATC. It is known that more than 90% of thyroid cancer harbor mutations in the MAPK pathway [48]. RAS mutations that occur both in benign and malignant thyroid cancers are detected also in ATCs, with variable frequency ranging from 6 to 50% of cases depending on series [49–53]. By contrast, RET/PTC rearrangements, which account for about 15–20% of PTCs, are rarely found in ATCs [47, 52]. Mutations in the BRAF gene, which occur in more than 50% of well-differentiated PTCs [54–58], are only detected in 25% of ATC cases [59, 60]. This lower frequency is in apparent contrast with the role of this mutation in driving aggressiveness of thyroid tumors, which has been proposed and largely debated in the past decade [61–63].

#### 3.1.1. PIK3CA

Gain of function mutations in the PIK3CA (phosphatidylinositol-4,5-bisphosphate 3-kinase, catalytic subunit alpha) gene is found in 25–40% of cancers, with alterations mainly clustering in two hotspots within the helical (exon 9) and catalytic (exon 20) domains [64]. In thyroid cancer, PIK3CA mutations are rare in PTCs (0–5% depending on series) but more frequent in poorly differentiated and anaplastic thyroid cancer (from 11 to 23%). As well, amplification of the PIK3CA genomic locus in 3q26.3 is found in about 40% of ATC suggesting that alteration of the PI3K depending pathway plays a pivotal role in the pathogenesis of ATCs [50, 51, 53, 65].

#### 3.1.2. TERT

Somatic mutations in the promoter of the TERT (Telomerase Reverse Transcriptase) gene have been described as highly recurrent in different types of cancer including thyroid cancer [66–69]. Up to 50% of ATCs (33–50%) have been shown to carry these mutations. Intriguingly, TERT promoter mutations seem to occur prevalently in those tumors harboring mutated BRAF or RAS, suggesting that TERT alteration is acquired later during tumor development and may provide a functional advance to BRAF or RAS-driven tumors by enabling acquisition of additional genetic defects leading to disease progression.

#### 3.1.3. CTNNB1

Wnt pathway appears also to play a relevant function in ATC development. Mutations in* CTNNB1 (β-Catenin)* gene leading to a constitutively active Wnt-signaling have been reported in 25–60% of ATCs [70]. CTNNB1 is a major component of the E-cadherin cell-cell adhesion complexes and a role of this protein in the epithelial-mesenchymal transition process has been demonstrated [71]. Intriguingly, the transdifferentiation of well-differentiated thyroid tumor cells toward a nondifferentiated status has been proposed as one of the major processes in the pathogenesis of ATCs.

#### 3.1.4. p53 and PTEN

Besides gain of function alterations in key oncogenes, tumor development and progression rely significantly on the inactivation of tumor suppressor genes. p53 and PTEN genes are involved in the negative regulation of cell proliferation and in promoting apoptosis and are frequently impaired during tumor progression. More than 50% of ATCs have been reported to carry loss of function mutations in the p53 gene. As well, the overexpression of p53, which may reflect altered function of the protein in the absence of mutation, has been frequently observed in ATC. Loss of function alterations in the PTEN gene, which inhibit the activation of the PI3K pathway, has been reported to occur in 4 to 16% of ATC [50, 51, 53].

### 3.2. Somatic Chromosomal Aberration

It is well established that the accumulation of genetic alterations is a driving mechanism of tumor growth and spread to distant sites. Several studies have investigated genomic instability and DNA copy number variations in ATC with the intent to understand the impact of genomic damage on the genesis and progression of this tumor. Liu and colleagues used real-time PCR analysis to investigate the copy number of a panel of genes involved in MAPK and PI3K pathway in thyroid cancer including a series of 51 ATCs. They observed that genes coding for tyrosine kinase receptors (RTK) like EGFR, PDGFR, VEGFR, KIT, and MET are frequently amplified in thyroid cancer and in particular in the ATC histotype [51]. Wreesman and colleagues used CGH technique to investigate the molecular-cytogenic profile of different histotypes of thyroid cancer to define chromosomic regions that could be specifically associated with the development of ATC [72]. These authors observed several chromosomal abnormalities that were common to both well-differentiated and nondifferentiated thyroid cancer (like gain of 5p15, 5q11–13, 19p, and 19q and loss of 8p) and that could represent early event in the genesis of these tumors. Furthermore, they found alterations like gain in 3p13-14, loss of 5q11–31, and gain in 11q13 that were exclusive of the genome of the 15 ATCs analyzed and that may represent late genetic events driving the transformation of a preexisting thyroid cancer into the aggressive ATC histotype. Using the same approach, Rodrigues et al. investigated the chromosomal profiles of 7 ATCs, showing that chromosomal imbalances affect the genome of all cases analyzed [73]. Intriguingly, the chromosomal regions affected by the alterations were extremely heterogeneous, suggesting the existence of a high-grade genetic interneoplastic diversity in ATCs. Besides chromosomal imbalances, Miura and colleagues reported that 6 out of 10 ATCs showed aneuploidy [74].

Summarizing the data currently available, two major considerations emerge: (1) the type of chromosomal alterations that characterize ATCs is widely heterogeneous and up to now it is not possible to define a “common” profile of alterations that is specific of this tumor type. This observation implies that different kinds of genetic damage may contribute to the genesis of this tumor. (2) ATCs are characterized by a higher number of chromosomal alterations with respect to well-differentiated and poorly differentiated thyroid cancer. However, several studies reported that the amount of genetic damage does not directly correlate with the grade of aggressiveness or the outcome of ATCs. Based on these considerations, we may hypothesize that the high-grade genomic instability observed in ATCs is a side effect of the loss of restraining mechanisms of cell proliferation rather than being the cause of tumor progression. Indeed, a number of mitotic proteins involved in cell cycle check points or engaged in chromosome assembly and segregation have been shown to be deranged in ATCs [75, 76]. These include the three members of the Aurora kinase family. Aurora kinases are implicated in several aspects of chromosome segregation and cytokinesis. Expression of all Aurora kinases and in particular of Aurora A is strongly induced in ATC cells [75, 76] and overexpression of Aurora A has been shown to induce centrosome amplification and to potentiate the oncogenic function of Ras [77, 78].

Evidences exist of a negative cross-talk between Aurora A kinase and p53 [79, 80]. Considering the fact that p53 is mutated or aberrantly expressed in a wide proportion of ATCs, it is likely to suppose that these alterations may affect the balance between p53 and Aurora A with relevant consequences on chromosome stability. The possibility to counteract the misfunctioning mitotic proteins has been considered a potential therapeutic strategy for cancer with high grade genetic damage. Indeed, inhibitors of Aurora kinases alone or in combination with other drugs, including microtubule inhibitors, showed an important anticancer effect in preclinical models of ATCs indicating this approach as a possible therapeutic strategy for ATCs treatment [75, 81].

## 4. Histogenesis

In literature there are indirect, although convincing, evidences that ATC represents a terminal dedifferentiation of preexisting well-differentiated thyroid carcinoma (WDTC) in most, if not all, cases. A large portion of ATC develops in longstanding goiters or in the context of preexisting, incompletely treated papillary or follicular thyroid cancers. Likewise, careful examination of primary ATC tumors reveals coexisting areas of WDTC in 80% to 90% of cases [82, 83]. This better differentiated tumor is usually a papillary carcinoma or one of its variants (particularly Warthin-like and tall cell variant), but it may also be a follicular carcinoma, as well as an oncocytic carcinoma, or an insular carcinoma [4, 26, 84, 85]. It has been suggested that if an extensive sampling is performed, foci of WDTC are eventually found in every specimen of ATC [83]. Furthermore, it has been postulated that the sharply outlined sclerohyaline nodules sometimes present within undifferentiated carcinoma represent the burn-out residue of such well-differentiated components [86].

**Figure 9:**
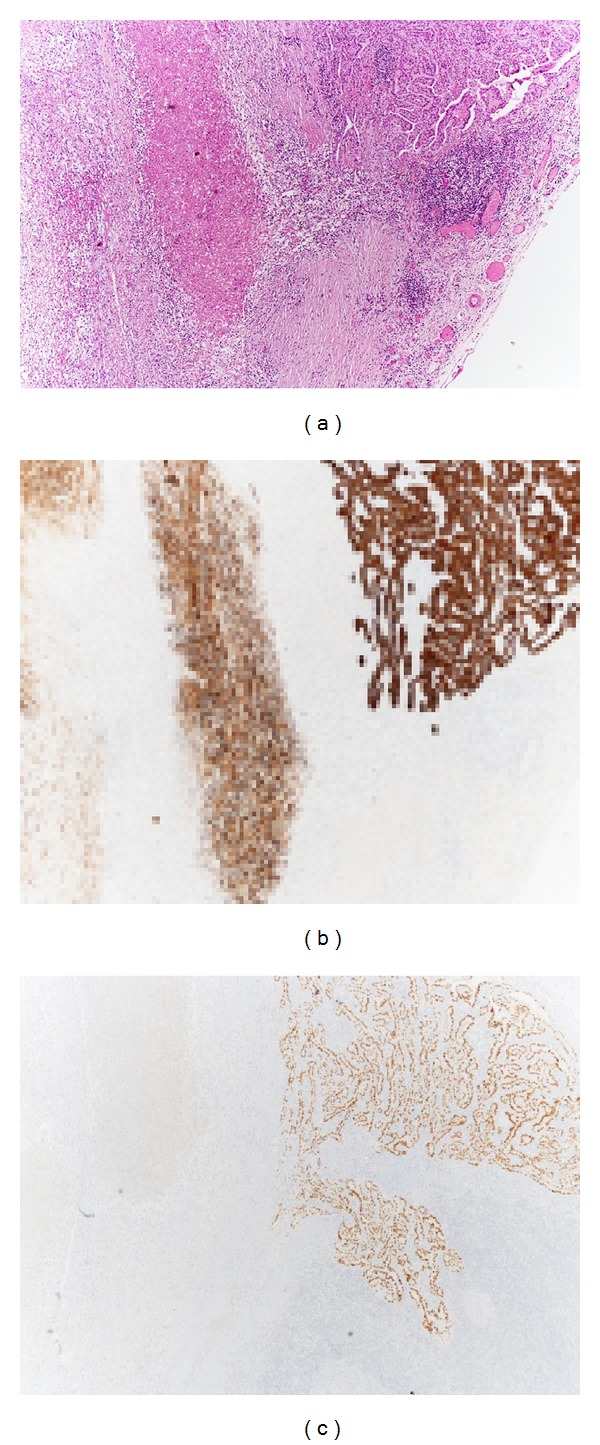
Lymph node metastasis of WDTC with anaplastic areas. Residual foci of papillary carcinoma are present in the right upper corner but the metastatic deposits are constituted mainly by spindle cells with wide necrotic areas (a). While both WDTC and ATC are immunoreactive with pankeratin (b), TTF1 is positive only in the WDTC (c).

Anaplastic transformation may also take place in a metastatic focus, (Figures 9(a), 9(b), and 9(c)) thus supporting the idea that these lesions originate through the dedifferentiation of preexisting well-differentiated cancer [87–89].

Nevertheless, according to the current genetic data it is conceivable that not all the ATCs arise as temporal aggressive evolution of a preexisting WDTC. If the ATC phenotype was always the temporal aggressive evolution of a preexisting WDTC then common founding alterations between the ATCs and the WDTC subgroups should be identified. Whole genome studies showed that the chromosomal asset of ATCs and WDPTC is widely different [51, 72–74] supporting the hypothesis that not all thyroid cancers start as indolent lesions but some of them may originate as already aggressive nondifferentiated cancer.

## Figures and Tables

**Table 1:** Differential diagnoses of sarcomatoid category.

Thyroid lesions simulating a sarcomatoid pattern		Differential features
*Other malignancies *	Primary or metastatic sarcoma	It is an exclusion diagnosis: (i) lack of a well-differentiated component; (ii) no epithelial markers; (iii) absence of palisading necrosis and neoplastic spindle cells infiltrating the wall of large-sized vessels; (iv) presence of extrathyroidal sarcoma clinically detected (in metastatic disease).
	SETTLE (spindle epithelial tumor with thymus-like elements)	(i) Adolescent or young adults (mean age 15 years); (ii) biphasic pattern of growth with a predominant spindle cell component merging with mucin-secreting glandular elements; both components have an epithelial phenotype; (iii) generally indolent behavior.
	Spindle cell variant of papillary thyroid carcinoma	Metaplastic variant of PTC: (i) spindle cells retain even if focally nuclear features of PTC; (ii) consistent immunoreactivity for thyroglobulin.
	Spindle cell variant of medullary carcinoma	(i) Presence of amyloid deposits; (ii) immunoreactivity for calcitonin and/or calcitonin gene-related peptide.

*Benign processes *	Solitary fibrous tumor	(i) Low mitotic rate (4 mitoses or fewer per 10 high-power fields); (ii) no necrosis or vascular invasion; (iii) positivity for bcl-2, CD34, CD99, and vimentin and negativity for all epithelial markers.
	Riedel thyroiditis	(i) Absence of necrosis; (ii) evidence of occlusive phlebitis (no angioinvasion); (iii) absence of neoplasm; (iv) negativity for epithelial markers; (v) generally benign self-limiting disease.
	Post-fine-needle aspiration Spindle cell nodules of the thyroid	(i) History of FNA biopsy; (ii) size ranging from 3 to 10 mm; (iii) not encapsulated but relatively circumscribed and located mostly in the center of preexisting thyroid nodules; (iv) low mitotic rate; (v) immunoreactivity for smooth muscle actin (myofibroblastic origin).

**Table 2:** Differential diagnoses of squamoid category.

Thyroid lesions simulating a squamoid pattern	Differential features
Pure squamous cells carcinoma	(i) Entirely composed of squamous cells; (ii) no evidence of another type of thyroid carcinoma in close proximity.

Primary head and neck squamous cell carcinoma	(i) Presence of in situ component in head and neck structures; (ii) features of “ab extrinseco” involvement of thyroid parenchyma; (iii) PAX8 is consistently negative.

Metastatic squamous cell carcinoma of the lung	(i) Presence of a lung nodule clinically detected; (ii) PAX8 is consistently negative.

Diffuse sclerosing variant of papillary thyroid carcinoma	(i) Abundant psammoma bodies; (ii) neoplastic cells retain nuclear features of PTC; (iii) immunoreactivity for thyroglobulin.

Mucoepidermoid carcinoma	(i) Combination of squamoid and mucinous components; (ii) thought to represent papillary carcinoma with extreme degree of squamous and mucinous metaplasia; (iii) low grade thyroid neoplasm.

Sclerosing mucoepidermoid carcinoma with eosinophilia	(i) Fibrohyaline stroma; (ii) striking infiltration of eosinophils; (iii) mucin secretion is often present; (iv) typically arising in Hashimoto thyroiditis (thought to derive from metaplastic squamous nests associated with inflammatory infiltrate).

CASTLE (carcinoma with thymus-like elements)	(i) Lymphoepithelioma-like carcinoma with foci of squamous differentiation; (ii) pushing margins; (iii) immunoreactivity for CD5, bcl-2, high molecular weight keratin, mcl-1 (thought to be ectopic thymic carcinoma arising from remnants of the branchial pouch); (iv) usually indolent behavior with tendency to late recurrences.

**Table 3:** Immunohistochemical features of ATCs.

Immunostain	Percentage of positive cases
Cytokeratin	40–100%
Vimentin	100% (in spindle cells)
EMA	30–50% (in squamoid cells)
CEA	Rarely (in squamoid cells)
Thyroglobulin	0% (false positivity due to nonneoplastic thyroid follicular cells entrapped in the tumor or diffusion from destroyed normal follicles)
TTF-1	0%
RET/PTC oncoprotein	0%
Calcitonin	0%
PAX8	0–79% of ATCs (probably depending on antibody used); 92% of ATCs with squamoid features

## References

[1] de Lellis RA, Lloyd RV, Heitz PU, Eng C (2004). *Pathology and Genetics of Endocrine Organs*.

[2] Carcangiu ML, Steeper T, Zampi G, Rosai J (1985). Anaplastic thyroid carcinoma: a study of 70 cases. *American Journal of Clinical Pathology*.

[3] Rosai J, Saxen EA, Woolner L (1985). Undifferentiated and poorly differentiated carcinoma. *Seminars in Diagnostic Pathology*.

[4] Venkatesh YS, Ordonez NG, Schultz PN, Hickey RC, Goepfert H, Samaan NA (1990). Anaplastic carcinoma of the thyroid. A clinicopathologic study of 121 cases. *Cancer*.

[5] Schmid KW, Kroll M, Hofstadter F, Ladurner D (1986). Small cell carcinoma of the thyroid. A reclassification of cases originally diagnosed as small cell carcinomas of the thyroid. *Pathology Research and Practice*.

[6] Us-Krašovec M, Golouh R, Auersperg M, Bešič N, Ruparčič-Oblak L (1996). Anaplastic thyroid carcinoma in fine needle aspirates. *Acta Cytologica*.

[7] Wan SK, Chan JKC, Tang SK (1996). Paucicellular variant of anaplastic thyroid carcinoma: a mimic of Riedel's thyroiditis. *American Journal of Clinical Pathology*.

[8] Canos JC, Serrano A, Matias-Guiu X (2001). Paucicellular variant of anaplastic thyroid carcinoma: report of two cases. *Endocrine Pathology*.

[9] Gaffey MJ, Lack EE, Christ ML, Weiss LM (1991). Anaplastic thyroid carcinoma with osteoclast-like giant cells: a clinicopathologic, immunohistochemical, and ultrastructural study. *The American Journal of Surgical Pathology*.

[10] Rosai J, Carcangiu ML, DeLellis RA (1992). *Tumors of the Thyroid Gland*.

[11] Carda C, Ferrer J, Vilanova M, Peydró A, Llombart-Bosch A (2005). Anaplastic carcinoma of the thyroid with rhabdomyosarcomatous differentiation: a report of two cases. *Virchows Archiv*.

[12] Rosai J, Carcangiu ML (1987). Pitfalls in the diagnosis of thyroid neoplasms. *Pathology Research and Practice*.

[13] Shin WY, Aftalion B, Hotchkiss E, Schenkman R, Berkman J (1979). Ultrastructure of a primary fibrosarcoma of the human thyroid gland. *Cancer*.

[14] Tanboonand J, Keskool P (2013). Leiomyosarcoma: a rare tumor of the thyroid. *Endocrine Pathology*.

[15] Tseleni-Balafouta S, Arvanitis D, Kakaviatos N, Paraskevakou H (1988). Primary myxoid chondrosarcoma of the thyroid gland. *Archives of Pathology & Laboratory Medicine*.

[16] Tong G, Hamele-Bena D, Liu JC, Horst B, Remotti F (2008). Fine-needle aspiration biopsy of primary osteosarcoma of the thyroid: report of a case and review of the literature. *Diagnostic Cytopathology*.

[17] Ryška A, Ludvíková M, Szépe P, Böör A (2004). Epithelioid haemangiosarcoma of the thyroid gland. Report of six cases from a non-Alpine region. *Histopathology*.

[18] Kaur A, Didolkar MS, Thomas A (2013). Angiosarcoma of the thyroid: a case report with review of the literature. *Endocrine pathology*.

[19] Eloy JA, Mortensen M, Gupta S, Lewis MS, Brett EM, Genden EM (2007). Metastasis of uterine leiomyosarcoma to the thyroid gland: case report and review of the literature. *Thyroid*.

[20] Kreze A, Zapotocka A, Urbanec T (2012). Metastasis of dermatofibrosarcoma from the abdominal wall to the thyroid gland: case report. *Case Reports in Medicine*.

[21] Hafez MT, Hegazy MA, Abd Elwahab K, Arafa M, Abdou I, Refky B (2012). Metastatic rhabdomyosarcoma of the thyroid gland, a case report. *Head and Neck Oncology*.

[22] Bauer TW, Rostock RA, Eggleston JC, Baral E (1984). Spindle cell carcinoma of the breast: four cases and review of the literature. *Human Pathology*.

[23] Leifer C, Miller AS, Putong PB, Min BH (1974). Spindle-cell carcinoma of the oral mucosa. A light and electron microscopic study of apparent sarcomatous metastasis to cervical lymph nodes. *Cancer*.

[24] Batsakis JG, Rice DH, Howard DR (1982). The pathology of head and neck tumors: spindle cell lesions (sarcomatoid carcinomas, nodular fasciitis, and fibrosarcoma) of the aerodigestive tracts, part 14. *Head & Neck Surgery*.

[25] Bronner MP, LiVolsi VA (1991). Spindle cell squamous carcinoma of the thyroid: an unusual anaplastic tumor associated with tall cell papillary cancer. *Modern Pathology*.

[26] Gopal PP, Montone KT, Baloch Z, Tuluc M, Livolsi V (2011). The variable presentations of anaplastic spindle cell squamous carcinoma associated with tall cell variant of papillary thyroid carcinoma. *Thyroid*.

[27] Dominguez-Malagon H, Flores-Flores G, Vilchis JJ (2001). Lymphoepithelioma-like anaplastic thyroid carcinoma: report of a case not related to epstein-barr virus. *Annals of Diagnostic Pathology*.

[28] Carr KA, Bulengo S, Weiss LM, Nickoloff BJ (1992). Lymphoepitheliomalike carcinoma of the skin: a case report with immunophenotypic analysis and in situ hybridization for Epstein-Barr viral genome. *The American Journal of Surgical Pathology*.

[29] Gulley ML, Amin MB, Nicholls JM (1995). Epstein-Barr virus is detected in undifferentiated nasopharyngeal carcinoma but not in lymphoepithelioma-like carcinoma of the urinary bladder. *Human Pathology*.

[30] Weinberg E, Hoisington S, Eastman AY, Rice DK, Malfetano J, Ross JS (1993). Uterine cervical lymphoepithelial-like carcinoma: absence of Epstein-Barr virus genomes. *American Journal of Clinical Pathology*.

[31] Toner M, Banville N, Timon CI (2014). Laryngotracheal presentation of anaplastic thyroid carcinoma with squamous differentiation: seven cases demonstrating an under-recognized diagnostic pitfall. *Histopathology*.

[32] Thompson LDR, Wieneke JA, Heffess CS (2005). Diffuse sclerosing variant of papillary thyroid carcinoma: a clinicopathologic and immunophenotypic analysis of 22 cases. *Endocrine Pathology*.

[33] Chan JKC, Rosai J (1991). Tumors of the neck showing thymic or related branchial pouch differentiation: a unifying concept. *Human Pathology*.

[34] Miyauchi A, Kuma K, Matsuzuka F (1985). Intrathyroidal epithelial thymoma: an entity distinct from squamous cell carcinoma of the thyroid. *World Journal of Surgery*.

[35] Bolat F, Kayaselcuk F, Nursal TZ (2007). Histopathological changes in thyroid tissue after fine needle aspiration biopsy. *Pathology Research and Practice*.

[36] LiVolsi VA, Brooks JJ, Arendash-Durand B (1987). Anaplastic thyroid tumors. Immunohistology. *The American Journal of Clinical Pathology*.

[37] Miettinen M, Franssila KO (2000). Variable expression of keratins and nearly uniform lack of thyroid transcription factor 1 in thyroid anaplastic carcinoma. *Human Pathology*.

[38] Ordonez NG, El-Naggar AK, Hickey RC, Samaan NA (1991). Anaplastic thyroid carcinoma: immunocytochemical study of 32 cases. *The American Journal of Clinical Pathology*.

[39] Quiros RM, Ding HG, Gattuso P, Prinz RA, Xu X (2005). Evidence that one subset of anaplastic thyroid carcinomas are derived from papillary carcinomas due to BRAF and p53 mutations. *Cancer*.

[40] Nonaka D, Tang Y, Chiriboga L, Rivera M, Ghossein R (2008). Diagnostic utility of thyroid transcription factors Pax8 and TTF-2 (FoxE1) in thyroid epithelial neoplasms. *Modern Pathology*.

[41] Fabbro D, Di Loreto C, Beltrami CA, Belfiore A, Di Lauro R, Damante G (1994). Expression of thyroid-specific transcription factors TTF-1 and PAX-8 in human thyroid neoplasms. *Cancer Research*.

[42] Tong G, Yu WM, Beaubier NT (2009). Expression of *PAX8* in normal and neoplastic renal tissues: an immunohistochemical study. *Modern Pathology*.

[43] Puglisi F, Cesselli D, Damante G, Pellizzari L, Beltrami CA, Di Loreto C (2000). Expression of Pax-8, p53 and bcl-2 in human benign and malignant thyroid diseases. *Anticancer Research*.

[44] Rivera M, Shang C, Gerhard R, Ghossein R, Lin O (2010). Anaplastic thyroid carcinoma: morphologic findings and PAX-8 expression in cytology specimens. *Acta Cytologica*.

[45] Bishop JA, Sharma R, Westra WH (2011). PAX8 immunostaining of anaplastic thyroid carcinoma: a reliable means of discerning thyroid origin for undifferentiated tumors of the head and neck. *Human Pathology*.

[46] Lee J, Hwang JA, Lee EK (2013). Recent progress of genome study for anaplastic thyroid cancer. *Genomics & Informatics*.

[47] Smallridge RC, Marlow LA, Copland JA (2009). Anaplastic thyroid cancer: molecular pathogenesis and emerging therapies. *Endocrine-Related Cancer*.

[48] Dhillon AS, Hagan S, Rath O, Kolch W (2007). MAP kinase signalling pathways in cancer. *Oncogene*.

[49] Fukushima T, Suzuki S, Mashiko M (2003). BRAF mutations in papillary carcinomas of the thyroid. *Oncogene*.

[50] Hou P, Liu D, Shan Y (2007). Genetic alterations and their relationship in the phosphatidylinositol 3-kinase/Akt pathway in thyroid cancer. *Clinical Cancer Research*.

[51] Liu Z, Hou P, Ji M (2008). Highly prevalent genetic alterations in receptor tyrosine kinases and phosphatidylinositol 3-kinase/Akt and mitogen-activated protein kinase pathways in anaplastic and follicular thyroid cancers. *Journal of Clinical Endocrinology and Metabolism*.

[52] Nikiforov YE (2004). Genetic alterations involved in the transition from well-differentiated to poorly differentiated and anaplastic thyroid carcinomas. *Endocrine Pathology*.

[53] Santarpia L, El-Naggar AK, Cote GJ, Myers JN, Sherman SI (2008). Phosphatidylinositol 3-kinase/Akt and Ras/Raf-mitogen-activated protein kinase pathway mutations in anaplastic thyroid cancer. *Journal of Clinical Endocrinology and Metabolism*.

[54] Cohen Y, Xing M, Mambo E (2003). BRAF mutation in papillary thyroid carcinoma. *Journal of the National Cancer Institute*.

[55] Gandolfi G, Sancisi V, Piana S, Ciarrocchi A (2014). Time to re-consider the meaning of BRAF V600E mutation in papillary thyroid carcinoma. *International Journal of Cancer*.

[56] Kimura ET, Nikiforova MN, Zhu Z, Knauf JA, Nikiforov YE, Fagin JA (2003). High prevalence of BRAF mutations in thyroid cancer: genetic evidence for constitutive activation of the RET/PTC-RAS-BRAF signaling pathway in papillary thyroid carcinoma. *Cancer Research*.

[57] Soares P, Trovisco V, Rocha AS (2003). BRAF mutations and RET/PTC rearrangements are alternative events in the etiopathogenesis of PTC. *Oncogene*.

[58] Xing M (2007). BRAF mutation in papillary thyroid cancer: pathogenic role, molecular bases, and clinical implications. *Endocrine Reviews*.

[59] Nikiforova MN, Kimura ET, Gandhi M (2003). BRAF mutations in thyroid tumors are restricted to papillary carcinomas and anaplastic or poorly differentiated carcinomas arising from papillary carcinomas. *Journal of Clinical Endocrinology and Metabolism*.

[60] Takano T, Ito Y, Hirokawa M, Yoshida H, Miyauchi A (2007). BRAFV600E mutation in anaplastic thyroid carcinomas and their accompanying differentiated carcinomas. *British Journal of Cancer*.

[61] Li C, Lee KC, Schneider EB, Zeiger MA (2012). BRAF V600E mutation and its association with clinicopathological features of papillary thyroid cancer: a meta-analysis. *Journal of Clinical Endocrinology and Metabolism*.

[62] Sancisi V, Nicoli D, Ragazzi M, Piana S, Ciarrocchi A (2012). BRAFV600E mutation does not mean distant metastasis in thyroid papillary carcinomas. *The Journal of Clinical Endocrinology & Metabolism*.

[63] Xing MM, Alzahrani AS, Carson KA (2013). Association between BRAF V600E mutation and mortality in patients with papillary thyroid cancer. *The Journal of the American Medical Association*.

[64] Samuels Y, Ericson K (2006). Oncogenic PI3K and its role in cancer. *Current Opinion in Oncology*.

[65] García-Rostán G, Costa AM, Pereira-Castro I (2005). Mutation of the PIK3CA gene in anaplastic thyroid cancer. *Cancer Research*.

[66] Heidenreich B, Rachakonda PS, Hemminki K, Kumar R (2014). TERT promoter mutations in cancer development. *Current Opinion in Genetics & Development*.

[67] Landa I, Ganly I, Chan TA (2013). Frequent somatic TERT promoter mutations in thyroid cancer: higher prevalence in advanced forms of the disease. *The Journal of Clinical Endocrinology and Metabolism*.

[68] Liu T, Wang N, Cao J (2013). The age- and shorter telomere-dependent TERT promoter mutation in follicular thyroid cell-derived carcinomas. *Oncogene*.

[69] Liu X, Bishop J, Shan Y (2013). Highly prevalent TERT promoter mutations in aggressive thyroid cancers. *Endocrine-Related Cancer*.

[70] Garcia-Rostan G, Tallini G, Herrero A, D'Aquila TG, Carcangiu ML, Rimm DL (1999). Frequent mutation and nuclear localization of *β*-catenin in anaplastic thyroid carcinoma. *Cancer Research*.

[71] Lamouille S, Xu J, Derynck R (2014). Molecular mechanisms of epithelial-mesenchymal transition. *Nature reviews Molecular Cell Biology*.

[72] Wreesmann VB, Ghossein RA, Patel SG (2002). Genome-wide appraisal of thyroid cancer progression. *The American Journal of Pathology*.

[73] Rodrigues RF, Roque L, Rosa-Santos J, Cid O, Soares J (2004). Chromosomal imbalances associated with anaplastic transformation of follicular thyroid carcinomas. *British Journal of Cancer*.

[74] Miura D, Wada N, Chin K (2003). Anaplastic thyroid cancer: cytogenetic patterns by comparative genomic hybridization. *Thyroid*.

[75] Isham CR, Bossou AR, Negron V (2013). Pazopanib enhances paclitaxel-induced mitotic catastrophe in anaplastic thyroid cancer. *Science Translational Medicine*.

[76] Ulisse S, Delcros JG, Baldini E (2006). Expression of Aurora kinases in human thyroid carcinoma cell lines and tissues. *International Journal of Cancer*.

[77] Miyoshi Y, Iwao K, Egawa C, Noguchi S (2001). Association of centrosomal kinase *STK15/BTAK* mRNA expression with chromosomal instability in human breast cancers. *International Journal of Cancer*.

[78] Tatsuka M, Sato S, Kitajima S (2005). Overexpression of Aurora-A potentiates HRAS-mediated oncogenic transformation and is implicated in oral carcinogenesis. *Oncogene*.

[79] Chen S, Chang PC, Cheng YW, Tang FM, Lin YS (2002). Suppression of the STK15 oncogenic activity requires a transactivation-independent p53 function. *The EMBO Journal*.

[80] Liu Q, Kaneko S, Yang L (2004). Aurora—a abrogation of p53 DNA binding and transactivation activity by phosphorylation of serine 215. *The Journal of Biological Chemistry*.

[81] Arlot-Bonnemains Y, Baldini E, Martin B (2008). Effects of the Aurora kinase inhibitor VX-680 on anaplastic thyroid cancer-derived cell lines. *Endocrine-Related Cancer*.

[82] Aldinger KA, Samaan NA, Ibanez M, Hill CS (1978). Anaplastic carcinoma of the thyroid: a review of 84 cases of spindle and giant cell carcinoma of the thyroid. *Cancer*.

[83] Nishiyama RH, Dunn EL, Thompson NW (1972). Anaplastic spindle-cell and giant-cell tumors of the thyroid gland. *Cancer*.

[84] Harada T, Ito K, Shimaoka K, Hosoda Y, Yakumaru K (1977). Fatal thyroid carcinoma. Anaplastic transformation of adenocarcinoma. *Cancer*.

[85] Lam KY, Lo CY, Wei WI (2005). Warthin tumor-like variant of papillary thyroid carcinoma: a case with dedifferentiation (anaplastic changes) and aggressive biological behavior. *Endocrine Pathology*.

[86] Chetty R, Mills AE, LiVolsi VA (1993). Anaplastic carcinoma of the thyroid with sclerohyaline nodules. *Endocrine Pathology*.

[87] Ozaki O, Ito K, Mimura T, Sugino K (1999). Anaplastic transformation of papillary thyroid carcinoma in recurrent disease in regional lymph nodes: a histologic and immunohistochemical study. *Journal of Surgical Oncology*.

[88] Al-Qsous W, Miller ID (2010). Anaplastic transformation in lung metastases of differentiated papillary thyroid carcinoma: an autopsy case report and review of the literature. *Annals of Diagnostic Pathology*.

[89] Nakayama R, Horiuchi K, Susa M (2012). Anaplastic transformation of follicular thyroid carcinoma in a metastatic skeletal lesion presenting with paraneoplastic leukocytosis. *Thyroid*.

